# Comparative Assessment of Viral Load Retention in Surgical and Fabric Masks Worn by COVID-19 Patients

**DOI:** 10.3390/v17121552

**Published:** 2025-11-27

**Authors:** Cristiane Monteiro Eller, Milena De Paula Rebello, Andreza Sálvio, Emanuelle S. R. F. Silva, Vinícius Silva Belo, Elba Regina E. Lemos, Marta Giovanetti, José Júnior França De Barros, Marco Aurélio Horta

**Affiliations:** 1 Biosafety Level 3 Facility (BSL-3), Instituto Oswaldo Cruz, Oswaldo Cruz Foundation, Rio de Janeiro 21040-900, Brazil; 2 Laboratory of Hantavirus and Rickettsias, Instituto Oswaldo Cruz, Oswaldo Cruz Foundation, Rio de Janeiro 21040-900, Brazil; 3 Laboratory of Virology and Molecular Parasitology, Instituto Oswaldo Cruz, Oswaldo Cruz Foundation, Rio de Janeiro 21040-900, Brazil; 4 Laboratory of Translational Neurosciences, Biomedical Institute, Federal University of the State of Rio de Janeiro, Rio de Janeiro 20211-040, Brazil; 5 COVID-19 Analytical Center, Instituto Oswaldo Cruz, Oswaldo Cruz, Rio de Janeiro 21040-900, Brazil; 6 Centro-Oeste Dona Lindu Campus, Federal University of São João del-Rei—UFSJ, Divinópolis 35501-296, Brazil; 7 Department of Sciences and Technologies for Sustainable Development and One Health, Universita Campus Bio-Medico di Roma, 00128 Rome, Italy; 8 Laboratory of Flaviviruses, Instituto Oswaldo Cruz, Oswaldo Cruz Foundation (Fiocruz), Rio de Janeiro 21040-900, Brazil

**Keywords:** SARS-CoV-2, face masks, viral load

## Abstract

Face masks are widely recognized as a key intervention to limit SARS-CoV-2 transmission, yet the distribution and persistence of viral RNA across different mask regions and layers remain poorly understood. To address this, we analyzed 185 masks collected from 60 SARS-CoV-2-positive individuals in Rio de Janeiro between December 2020 and September 2022. Masks were sectioned into anatomical regions (nose, mouth, sides) and structural layers (inner, middle, outer), and viral RNA was quantified using RT-qPCR. Samples with the highest viral loads were selected for partial sequencing of the spike gene, and paired analyses with swab samples were performed. Statistical comparisons included non-parametric tests and a linear mixed-effects model. Our results showed that the inner layer and nose region consistently harbored the highest viral RNA levels, with no significant differences between surgical and fabric masks. Viral load decreased by an estimated 39% per day, consistent with exponential decay. Sequencing confirmed identical viral genomes in masks and swabs and allowed identification of circulating variants, including Gamma and Omicron. These findings indicate that masks serve not only as effective physical barriers but also as non-invasive sources for genomic surveillance, providing insights into viral shedding patterns and informing strategies for monitoring and controlling SARS-CoV-2 transmission.

## 1. Introduction

COVID-19 first emerged in December 2019 in Wuhan, China, rapidly spreading throughout the country and subsequently reaching other parts of Asia, Europe, and the Americas. On 30 January 2020, the World Health Organization declared the outbreak a Public Health Emergency of International Concern, and by 11 March 2020, it was officially classified as a pandemic [1,2].

SARS-CoV-2 is a positive-sense single-stranded RNA virus that encodes several structural proteins, including spike (S), envelope (E), membrane (M), and nucleocapsid (N). The spike glycoprotein, located on the viral surface, contains a receptor-binding domain (RBD) that facilitates entry into host cells by binding to the human angiotensin-converting enzyme 2 (ACE2) receptor [3]. This protein is both antigenic and immunogenic, playing a key role in eliciting host immune responses and enabling vaccine evasion [4,5]. Variants of Concern (VOCs)—including Gamma, Delta, and Omicron—are characterized by mutations that enhance transmissibility or disease severity, or contribute to immune escape. These genetic changes may reduce the effectiveness of vaccines and therapeutics, underscoring the need for continuous genomic surveillance [6].

Initially regarded as a respiratory illness, COVID-19 is primarily transmitted via airborne particles. Most infections are asymptomatic or mild, resembling upper respiratory tract infections, though asymptomatic individuals remain capable of transmitting the virus. Moderate to severe cases may require hospitalization, and fatalities were more common before the widespread rollout of vaccination campaigns [5,7]. The incubation period typically ranges from 5 to 6 days but can extend up to 14 days, during which transmission can occur despite the absence of symptoms [8].

The pandemic underscored the importance of understanding transmission mechanisms and implementing effective mitigation strategies. Given that respiratory droplets and aerosols are the primary transmission routes, face masks emerged as a critical public health intervention. Masks reduce the emission of viral particles into the environment and protect the wearer by filtering infectious aerosols [9,10,11]. Their efficacy depends on several factors, including mask type, fit, material, and proper usage [12].

As the pandemic escalated, particularly in resource-limited settings, shortages of surgical masks led to the widespread use of cloth face coverings [8,13,14]. While all mask types provide some degree of protection, cloth masks are generally less effective than surgical or N95 masks, particularly when the latter are properly fitted [15,16]. The filtration efficiency of cloth masks varies significantly based on fabric type and construction. Materials such as linen and silk offer limited protection, whereas denser fabrics like felted wool or tightly woven cotton are more effective [17,18]. Additionally, mask efficacy is substantially higher when worn by infected individuals, thereby reducing viral emission at the source [19].

In addition to preventing infectious disease transmission, face masks have demonstrated protective effects against environmental hazards, such as wildfire smoke, contributing to reductions in related hospital admissions [20,21]. The effectiveness of cloth masks depends on several variables, including fabric composition, number of layers, fit, and hygienic practices, which are essential for safe reuse [22].

Despite advances in vaccination and declining case numbers, face masks remain a key tool in reducing COVID-19 transmission and were among the most widely adopted public health measures during the pandemic. It has been observed that masks worn for prolonged periods by infected individuals accumulate viral RNA, which may reflect the infectious status of the wearer [23]. Previous studies have also demonstrated that SARS-CoV-2 RNA can persist on various environmental surfaces, including in specific locations around patients in clinical settings, indicating that spatial distribution plays a role in viral persistence and even in masks worn by the patients [24]. However, there is limited information regarding how viral particles distribute across distinct regions and layers of face masks. To our knowledge, no studies to date have systematically addressed this aspect, making our investigation one of the first to explore this dimension of viral contamination in masks.

This study aimed to expand the current understanding of SARS-CoV-2 contamination in face masks used by infected individuals. Specifically, it investigates the distribution of viral RNA across various mask regions (nose, mouth, sides) and layers (inner, middle, outer), compares viral retention between surgical and fabric masks, and assesses the persistence of viral RNA over time. Additionally, the study explored the feasibility of conducting genomic sequencing directly from used masks, thereby contributing to public health surveillance strategies.

## 2. Materials and Methods

### 2.1. Study Population and Sample Collection

Between December 2020 and September 2022, nasal swab samples and face masks were collected from individuals suspected of SARS-CoV-2 infection in Rio de Janeiro, Brazil—a country with over 210 million inhabitants and a high COVID-19 burden. Collections occurred at the Teatro Municipal, where artistic staff had resumed partial in-person work, and at the Instituto Benjamin Constant, which serves visually impaired individuals and maintained limited on-site activities. Participants were approached during institutional testing; those with positive rapid antigen test results were classified as suspected COVID-19 cases and invited to participate in the study, providing additional material for RT-qPCR and viral load analysis. Most agreed after receiving information about the project. In addition, a subset of self-referred individuals—symptomatic or exposed—who were aware of the study voluntarily sought testing and were included following the same procedures.

All collections were conducted based on medical recommendations and with informed consent. Nasopharyngeal swabs were collected by inserting a swab into each nostril until resistance was met (either the inferior turbinate or nasopharyngeal cavity), followed by rotation and removal. Swabs were placed in 3 mL of the viral transport medium (VTM; Xpert nasopharyngeal sample collection kit, Cepheid, Sunnyvale, CA, USA).

Participants who tested positive in the rapid antigen test were asked to provide the face masks they were wearing at the time of testing and to store subsequently used masks in home freezers for future retrieval. Institutional vehicles facilitated home visits to collect masks and swabs over time, but due to the lockdown imposed by the authorities, the monitoring was performed when possible. A total of 185 masks from 60 individuals were analyzed; 26 of these individuals submitted follow-up samples when possible. For partial Spike gene sequencing, only paired masks and swab samples with cycle threshold (Ct) values below 30 (corresponding to >6.44 × 10^5^ copies/mL) were selected, resulting in a subset of 12 participants.

All collected masks were classified as either surgical or fabric and were sectioned into three main regions—nose, mouth, and sides (left and right). Multilayer masks were further separated into inner, middle, and outer layers. Basic demographic data, including sex and age, were recorded.

### 2.2. Processing of Masks and Swabs

**Figure 1:**
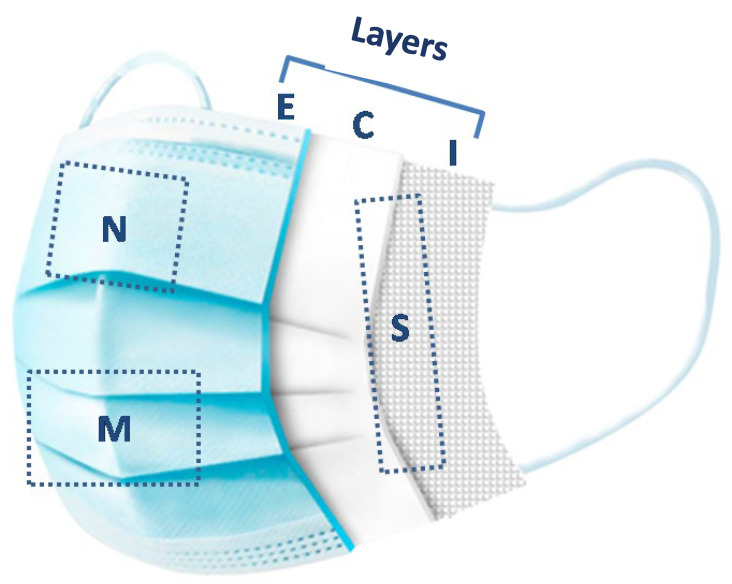
Locations where the cuts were made on the masks, N—Nose, M—Mouth, and S—side, and the view of the different layers, I—internal, C—central, and E—External.

Nasopharyngeal swab samples were resuspended in 3 mL of VTM immediately after collection. Mask samples were cut as follows: 2 cm wide full height with seams removed for the sides (left side and right side); 5 cm × 5 cm for the nose (N); and 5 cm × 8 cm for the mouth (M). Because facial anatomy and mask fit vary among individuals, airflow trajectories and contact surfaces also differ, influencing the sites where viral particles accumulate. Although these person-to-person variations could not be controlled under real-world conditions, all masks were sectioned according to standardized anatomical reference points (nose, mouth, right and left sides) to ensure consistent regional comparison across participants. Each region was submerged in VTM (Figure 1). For multi-layered masks, each region was separated into internal (I), central (C), and external (E) layers, following the previously mentioned dimensions. The mask part with the lowest Ct value from the four main areas (mouth, nose, left side, and right side) was selected for analysis, and only these results are presented in the table. Samples were incubated at 4 °C for at least 30 min to 12 h. After vortexing, the VTM was transferred into labeled 1.5 mL tubes using a 2 mL Pasteur pipette, and swabs or masks were discarded. All samples were stored at −80 °C.

### 2.3. SARS-CoV-2 Molecular Detection

Nucleic acids were extracted from 300 µL of each sample using the DNA/RNA 300 kit H96 and Janus G3/Janus Chemagic automated extraction systems (Perkin-Elmer, Waltham, MA, USA). The Janus 360 system uses magnetic beads to extract viral nucleic acids, following the manufacturer’s instructions. The final volume of the sample was 50 µL. SARS-CoV-2 RNA was detected using a qRT-PCR assay targeting the E gene (Bio-Manguinhos, Rio de Janeiro, Brazil) [23], prepared using the Janus G3 system (Perkin-Elmer, Waltham, MA, USA), according to the manufacturer’s protocol. Quantification was performed by quantitative reverse-transcription PCR (qRT-PCR) using an in-house single-stranded RNA standard curve (Limit of Detection: 599 copies/mL). The assay employed a FAM-labeled probe for the E gene and a VIC-labeled probe for the human RNase P (RP) gene (internal control). The positive and negative controls provided with the kit were included in every run to ensure assay reliability.

Samples were considered positive for the E region if the Ct value was below 38.0 (2.42 × 10^3^ copies/mL). Samples with a Ct value above 38.0 or undetectable Ct values were considered negative. For the RP gene, a Ct value of 35.0 (1.96 × 10^4^ copies/mL) or lower validated the experiment. A Ct value for the positive control below 37.0 (4.86 × 10^3^ copies/mL) was required to validate the assay. For samples showing a Ct value around 38 (2.42 × 10^3^ copies/mL) had their results repeated for confirmation. Only samples showing detectable RNA copies in both the initial and repeat analyses were considered positive. Data from these trials, along with participant information, were entered into a database for further analysis and comparison. To rule out co-contamination, all nasopharyngeal swab samples were also tested for Influenza A and B. No positive results were detected.

### 2.4. Amplification and Sequencing of the Spike Protein Gene

To amplify the region encoding part of the SARS-CoV-2 S protein gene, two sets of gene-specific primers were used in a two-step procedure, as previously described [24]. This involved two reactions: RT-PCR followed by PCR. The RT-PCR was performed using the Super Script^®^ IV One Step RT-PCR System with Platinum SuperFi RT-PCR Master Mix (Invitrogen, Carlsbad, CA, USA), with sense and antisense oligonucleotides (Table 1) at a concentration of 10 pmol/μL. Thermocycling conditions were as follows: cDNA synthesis at 45 °C for 10 min and 98 °C for 2 min. This was followed by 35 cycles of 98 °C for 10 s, 58 °C for 10 s, and 72 °C for 1 min, with a final extension at 72 °C for 5 min.

**Figure 2:**
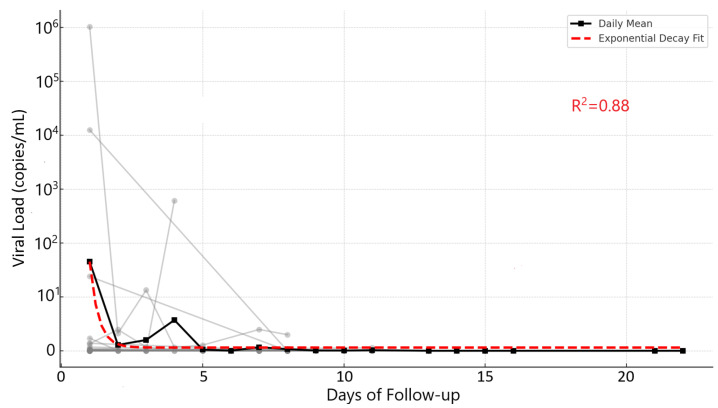
Individual viral load trajectories (gray lines) and daily mean viral load (black squares) in 93 face mask samples collected over time from 25 individuals. The dashed red line represents the fitted exponential decay model (R^2^ = 0.88) describing the overall trend in viral load reduction.

The resulting amplicon (1719–1728 bp) was used as the template for the second, nested PCR using the same oligonucleotide concentration (10 pmol/μL) and an additional set of internal primers (Table 1 and Figure 2). This amplification was carried out using Phusion Hot Start II DNA Polymerase with GC Buffer (Thermo Fisher Scientific, Waltham, MA, USA). Cycling conditions included an initial denaturation at 98 °C for 30 s, followed by 45 cycles at 98 °C for 10 s, 62 °C for 30 s, and 72 °C for 1 min, concluding with a final extension at 72 °C for 10 min. The final product (1583–1592 bp) was visualized on 1.5% agarose gel (Sigma-Aldrich, St. Louis, MO, USA) prepared in Tris-Borate-EDTA (TBE) buffer (89 mM Tris-borate, 2 mM EDTA, pH 8.3), electrophoresed at 80 W/h, and photo-documented using a transilluminator (STi gel, Loccus Biotecnologia, Diadema, SP, Brazil). The PCR product was diluted in bromophenol blue, stained with GelRed^®^ 10,000× (Biotium Inc., Fremont, CA, USA) at a working concentration of 20× (1:500) and estimated using a 1 kb Plus molecular marker (Invitrogen/Life Technologies, Carlsbad, CA, USA).

Purification of the PCR product was conducted using the QIAquick PCR Purification Kit (Qiagen, Hilden, Germany), and quantification was estimated with a molecular low-mass marker (Invitrogen/Life Technologies, Carlsbad, CA, USA). Amplicons were sequenced using the Sanger method, employing the same primers that flank the second round along with internal primers to ensure bidirectional coverage of the entire amplified region (Table 1). Sequencing was performed with the Big Dye Terminator Cycle Sequencing Ready Reaction Kit (Applied Biosystems, Foster City, CA, USA) on an ABI Prism 3730 Genetic Analyzer (Applied Biosystems, Foster City, CA, USA). The final assembled fragment of the spike gene comprised 1,549 base pairs (bp), covering a continuous region of the S gene with bidirectional coverage. Sequence alignment, editing, and analysis of nucleotide sequences identifying mutations, variants, and deduced amino acid sequence similarities of the S gene were performed using MEGAX v12 and Bio Edit v7.0.5 programs. The Wuhan strain (NC_045512.2, gen bank accession number) was used as a reference to align and edit sequences. Sequences were typed using the Genome Detective 1·126 (https://www.genomedetective.com) and Nextclade program (https://clades.nextstrain.org, accessed on 10 July 2025) and a Basic Local Alignment Search Tool (BLAST) (https://blast.ncbi.nlm.nih.gov/Blast.cgi accessed on 1 October 2025). All sequences have been submitted to GISAID (https://www.gisaid.org/ accessed on 5 October 2025) and to GenBank under the accession numbers PV797855, PV797856, PV797857, PV797858, PV797859, PV797860, PV797861, PV797862, PV797863, PV797864, PV797865, and PV797866.

### 2.5. Statistical Analysis

Viral load data from 185 masks were analyzed across anatomical regions, layers, and mask types. The Kruskal–Wallis test assessed differences in viral loads between the nose, mouth, and side regions. If significant, pairwise Mann–Whitney U tests with Bonferroni correction were applied. Mann–Whitney U tests compared viral loads between surgical and fabric masks across different layers. Statistical significance was set at *p* < 0.05.

A subset of participants provided additional masks collected on different days of infection. These samples were self-stored at home in domestic freezers and retrieved during home visits by the research team when logistics permitted. Because of lockdown restrictions and limited vehicle availability, follow-up collections were irregular and occurred on non-consecutive days, depending on participant availability and mask storage. No strict guidelines were imposed regarding mask use duration, though participants were instructed to store masks used for several hours per day. For longitudinal analysis, only masks from individuals who had stored masks across multiple days were considered. The day of the first RT-qPCR-positive swab collection was designated as Day 1. Subsequent masks were included based on the availability of stored material, even if not collected on consecutive days.

To evaluate temporal trends in viral load, an exponential decay model was fitted to the daily values available for everyone. In addition, a linear mixed-effects model (LMM) was applied to account for repeated measures and between-subject variability over time. This approach allowed the estimation of overall decay patterns despite the lack of uniform sampling intervals across participants.

## 3. Results

### 3.1. Descriptive Analysis of Viral Load in Masks

Analysis of viral load distribution across different mask regions (nose, mouth, and sides) and layers (internal, central and external) revealed distinct patterns. The highest mean viral load was detected in the nose region (2.43 × 10^6^ ± 1.93 × 10^7^ copies/mL), followed by the mouth region (1.17 × 10^6^ ± 9.28 × 10^6^ copies/mL), with the lowest levels observed at the sides (7.07 × 10^4^ ± 7.17 × 10^5^ copies/mL). Regarding mask layers, the internal layer consistently harbored the highest viral load compared to the middle and outer layers (Table 2).

### 3.2. Comparison of Viral Load Among Different Mask Regions

The Kruskal–Wallis test revealed no significant difference in viral load among the three mask regions (*p* = 0.09). Pairwise Mann–Whitney U tests showed no significant difference between the nose and mouth regions (*p* = 0.52) or between the mouth and sides (*p* = 0.13). However, the nose region exhibited a significantly higher viral load compared to the sides (*p* = 0.03), suggesting a greater concentration of viral particles in areas directly associated with respiratory emission.

### 3.3. Comparison of Viral Load Among Mask Layers

In the nose region, the internal layer exhibited a significantly higher viral load than the middle layer (*p* < 0.05). The difference between the internal and external layers approached statistical significance (*p* = 0.05), while no significant difference was found between the middle and external layers (*p* = 0.73). In the mouth region, the internal layer had a significantly higher viral load than both the middle (*p* < 0.001) and external layers (*p* < 0.01). No significant difference was observed between the middle and external layers (*p* = 0.46). In the side region, no statistically significant difference in viral load was found between the right and left sides of the mask (*p* = 0.46). These findings reinforce the observation that the internal mask layer, being in direct contact with exhaled air, accumulates the highest viral load.

### 3.4. Comparison Between Surgical and Fabric Masks

No statistically significant differences in viral load were observed between surgical and fabric masks in any of the nasal layers (intern: *p* = 0.85, center: *p* = 0.33, extern: *p* = 0.77) (Table 3). Although surgical masks exhibited higher mean viral loads across all nasal layers; the differences were not statistically significant. Fabric masks demonstrated lower median values and narrower distributions. Similarly, in the mouth region, no significant differences were detected between mask types in any layer (intern: *p* = 0.72, center: *p* = 0.32, extern: *p* = 0.76). Surgical masks showed higher average viral loads in the intern and center layers, whereas fabric masks exhibited slightly higher means in the external layer. Despite these numerical differences, statistical analysis confirmed no significant variation between mask types.

### 3.5. Longitudinal Analysis of Viral Load Reduction

To evaluate the persistence of viral contamination on masks over time, a longitudinal analysis of individual viral load trajectories was conducted. Overall, the viral load levels declined during the follow-up period, although some participants exhibited transient fluctuations (Figure 2). The model demonstrated a strong fit to the data (R^2^ = 0.88), supporting a consistent downward trend. Visual inspection of participant curves (gray lines) further confirmed this pattern, despite variations between individuals.

Each additional day of follow-up was associated with an average reduction of approximately 0.216 log_10_ units in viral load (*p* < 0.001), corresponding to an estimated 39% daily reduction, suggesting a strong temporal decay pattern. The LMM also identified significant inter-individual variability (random intercept variance = 1.44, *p* < 0.001).

### 3.6. Analysis of the Partial Sequencing of the Spike

To assess the circulation of variants of interest within the study cohort over time, individuals with the highest viral loads in both nasopharyngeal swab and mask samples were selected to maximize the likelihood of successful sequencing. A total of 24 samples met these criteria (12 from swabs and 12 from masks). However, amplification failed in three swab samples and nine mask samples, resulting in successful sequencing of nine swab samples and three corresponding mask samples.

**Figure 3:**
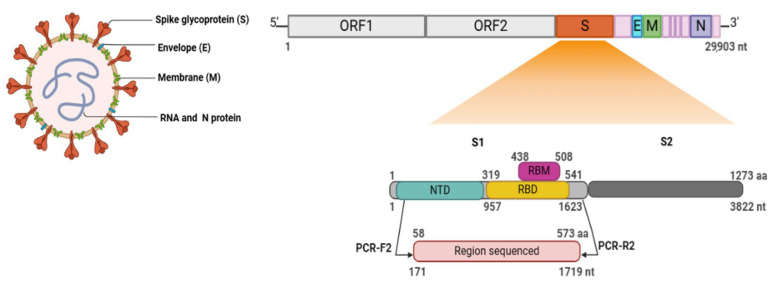
Schematic representation of the complete SARS-CoV-2 virion and the genomic organization of the spike (S) gene. The viral particle illustration highlights the structural proteins: spike glycoprotein (S), envelope (E), membrane (M), and nucleocapsid (N). The genomic map of the spike gene shows the S1 and S2 subunits, including the N-terminal domain (NTD), receptor-binding domain (RBD), and receptor-binding motif (RBM). Amino acid (aa) and nucleotide (nt) positions correspond to the Wuhan reference strain (NC_045512.2). The region amplified and sequenced in this study is shown in red, with primer binding sites indicated (PCR-F2 and PCR-R2). Colors used in the figure correspond to the functional domains defined above. Created in BioRender. Rebello, M. (2025) https://BioRender.com/wdvt4i7.

**Figure 4:**
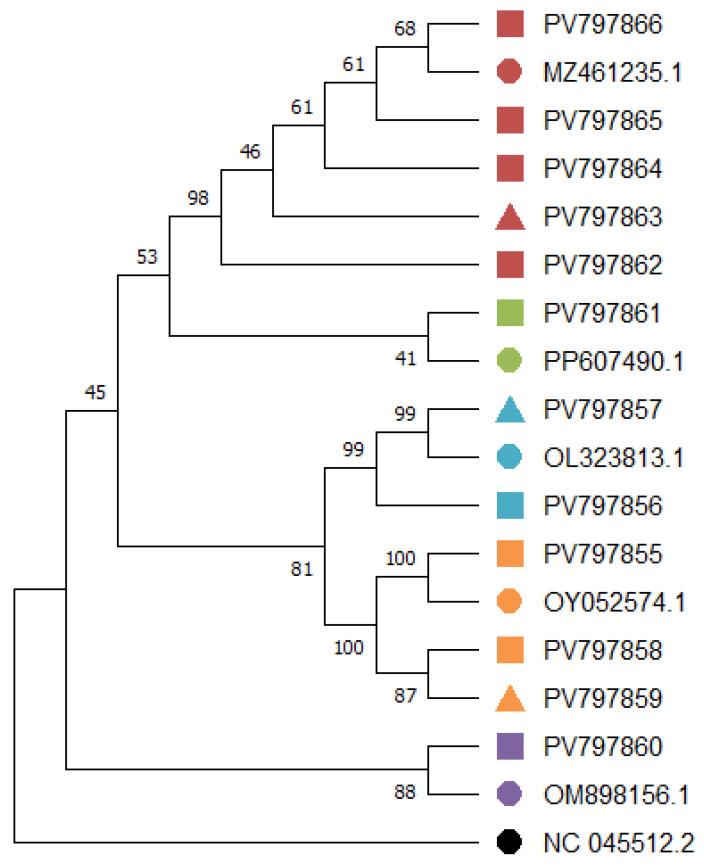
Neighbor-Joining phylogenetic tree based on partial SARS-CoV-2 Spike RBD sequences obtained from nasopharyngeal swabs and face mask samples. The tree was inferred using the p-distance (Hamming) model in MEGA v.12 and rooted using the ancestral Wuhan strain (NC_045512.2). Node support values were calculated with 1000 bootstrap replicates and are shown at each branch. Sample types are indicated by symbols (▲ mask samples; ■ swab samples), while reference sequences appear as ⯃. Colors represent major SARS-CoV-2 lineages detected in this study (Gamma—red, Delta—blue, Omicron—orange, 19B—purpure, 20B—green and Wuhan—black).

Genetic analysis revealed that the viral RNA recovered from each mask was identical to the respective swab from the same individual, confirming the reliability of mask-based sampling. Sequencing targeted most of the N-terminal domain (NTD) and the complete receptor-binding domain (RBD) within the S1 region of the S gene (Figure 3), which are key for variant classification. Among the nine individuals sequenced, five were infected with the Gamma variant, three with Omicron, and two with Delta. Two additional sequences did not match any variant of interest or concern (VOI/VOC) but were classified within clades 19B and 20B, respectively, based on their mutation and deletion profiles (Appendix A and Figure 4). These sequences did not exhibit hallmark mutations of VOCs, including N501Y, K417N/T, or L452R, supporting their designation as ancestral or early pandemic lineages. 

As expected, the Gamma and Omicron sequences displayed key mutations in the RBD region associated with increased transmissibility (e.g., N501Y) and immune escape (e.g., E484K/A, K417N/T). In contrast, the 19B and 20B clade sequences retained ancestral genomic features and lacked critical spike mutations characteristic of later-emerging variants, such as those associated with enhanced receptor binding or immune evasion. Key epidemiological characteristics of individuals whose samples underwent sequencing are presented in Appendix A; it also presents the variants of concern, if the sequence presents any deletions and the amino acid substitutions when present (in red). ID: Sample identification; A—Alanine; E—Glutamic Acid; K—Lysine; N—Asparagine; T—Threonine; R—Arginine; L—Leucine; and Y—Tyrosine.

## 4. Discussion

This study provides novel insights into the spatial distribution and retention of SARS-CoV-2 RNA in face masks worn by infected individuals. Our findings confirm that the inner mask layer, particularly in the nasal and mouth regions, retains the highest viral loads, which aligns with the known physiology of viral exhalation and deposition. These results reinforce the role of masks as effective barriers at the source of emission and contribute to the understanding of how respiratory particles are captured during active infection. Moreover, the absence of significant differences between surgical and fabric masks in terms of RNA retention highlights that both mask types offer comparable protective performance, particularly when properly used. By analyzing the decay of viral load over time and successfully sequencing viral genomes directly from used masks, this work also supports the feasibility of using masks not only as protective devices but as tools for non-invasive genomic surveillance.

The observed inter-individual variability in viral load retention across mask regions and over time may reflect a combination of biological and behavioral factors, including differences in viral shedding, respiratory patterns, and immune responses. It is also plausible that vaccination status influenced viral dynamics, as prior studies have shown that vaccinated individuals tend to exhibit reduced viral loads and shorter periods of viral shedding [26]. Although vaccination data were not collected in this study, it is noteworthy that approximately one-third of the participants were infected before Brazil’s national immunization campaign began in January 2021, suggesting that part of the cohort was likely unvaccinated at the time of infection. This temporal context may help explain the heterogeneity observed in viral load kinetics and highlights the importance of integrating epidemiological background when interpreting viral transmission data.

Regarding viral load distribution, our findings align with previous research on respiratory particle deposition. Exhaled droplets and aerosols are primarily expelled through the nose and mouth, leading to higher viral accumulation in these areas [27,28,29]. Our results confirm this pattern, demonstrating significantly higher viral loads in the nose and mouth regions compared to the sides of the mask. The lower viral load detected on the sides suggests minimal lateral dispersion, reinforcing the understanding that masks are most effective in preventing forward transmission [29,30]. The slightly elevated contamination on the right side may be attributed to more frequent manipulation of the mask by right-handed individuals, underscoring the importance of educating the public on proper mask handling to minimize self-contamination. From a prevention perspective, these results also highlight the need for educational strategies focused on mask hygiene. Additionally, for reusable masks, such findings support recommendations for daily washing and the importance of correct mask storage to prevent secondary exposure or transmission.

Consistently, the internal mask layer of the mask exhibited the highest viral load across all tested regions, emphasizing its role in capturing respiratory droplets upon exhalation. This observation aligns with previous studies demonstrating that the inner mask layer is the primary barrier for viral retention due to its direct exposure to exhaled breath [19,23]. Furthermore, the absence of statistically significant differences in viral load between surgical and fabric masks suggests that both types provide comparable protection when used correctly, supporting earlier research on mask filtration efficiency [29,31,32]. These findings underscore the importance of maintaining mask hygiene, as viral accumulation on the internal layer could contribute to self-inoculation if reused without adequate cleaning [33].

Although numerical differences in viral load were observed between surgical and fabric masks, no statistically significant advantage was identified in viral retention. While some studies report that surgical masks offer superior filtration efficiency [16], others have demonstrated that multi-layered fabric masks perform comparably in real-world settings [34]. These findings also suggest that fit and material composition play a crucial role in determining protective efficacy. Overall, these findings reinforce the role of masks in reducing viral transmission, particularly by limiting forward dispersion, and highlight the necessity of continued mask usage in high-risk environments.

The decline in viral load over time observed in our study is consistent with established models of viral clearance in respiratory infections, where viral shedding progressively diminishes as the immune response intensifies [29,35]. Our data demonstrated a 39% average daily reduction in viral load, which aligns with previous reports on SARS-CoV-2 decay kinetics in nasopharyngeal and environmental samples [36]. Notably, the LMM revealed significant inter-individual variability, reflecting differences in host immunity, viral replication dynamics, and behavioral factors such as mask usage and exhalation patterns [37]. These findings reinforce the importance of time-dependent viral decay in infection control strategies, as viral load reduction correlates with lower transmission risk over time.

The successful sequencing of viral genomes from both face masks and their corresponding nasopharyngeal swabs provides strong evidence that masks can serve as effective tools for genomic surveillance. The genetic concordance between mask-derived and swab-derived viral sequences rules out external contamination and validates the utility of mask sampling for variant detection. While previous studies have demonstrated the feasibility of recovering viral RNA from environmental surfaces and personal protective equipment samples [23,38,39], our findings extend this evidence by confirming that masks can retain intact viral genetic material suitable for sequencing. This opens possibilities for implementing non-invasive surveillance approaches, particularly in community-wide screening or resource-limited healthcare environments.

In our cohort, the detection of Gamma, Delta, and Omicron variants aligns with the known epidemiological landscape of SARS-CoV-2 in Brazil during the study period [40,41,42,43]. The identification of key RBD mutations such as N501Y, L452R, K417N/T, and E484K/A—which are associated with increased transmissibility and immune escape [44,45,46]—reflects the adaptive evolution of the virus and its potential to overcome prior immunity, with direct implications for vaccine effectiveness and reinfection risk.

Importantly, two sequences did not cluster within recognized Variants of Concern (VOCs) or Variants of Interest (VOIs) but were classified as belonging to clades 19B and 20B, respectively—lineages associated with early pandemic phases. These sequences lacked the full set of defining mutations typical of more recent variants but presented noteworthy features. In particular, the sequence PV797861, although assigned to clade 20B, harbored the E484K mutation—a substitution commonly found in Beta (B.1.351) and Gamma (P.1) variants. The isolated presence of E484K, in the absence of other hallmark mutations of those VOCs, suggests a possible homoplastic event or an independent adaptive mutation arising within a 20B genetic background. Given that the classification was based on a partial sequence, the conservative assignment to clade 20B remains appropriate; however, it highlights how convergent evolution can lead to immune escape traits even in early viral lineages.

Additionally, one of the sequences classified as Omicron did not exhibit the L452R mutation, which is a defining feature of certain Omicron sublineages, including BA.4 and BA.5. This absence may indicate that the sequence belongs to an earlier Omicron sublineage such as BA.1, or that it represents intra-lineage diversity possibly due to region-specific evolutionary pressures. Alternatively, it may reflect sequencing limitations inherent to partial genomic coverage. Nonetheless, the lack of this mutation within an otherwise Omicron-classified sequence reinforces the heterogeneity and ongoing diversification within major SARS-CoV-2 lineages, emphasizing the need for continued monitoring of variant-defining mutations even within established classifications.

These findings reinforce the importance of continued genomic surveillance using diverse sampling strategies. By capturing viral particles directly from the source of exhalation, face masks not only serve as physical barriers against transmission but also act as passive collectors of viral genomes—offering a complementary and non-invasive strategy for monitoring the spread and evolution of SARS-CoV-2. Moreover, the detection of mutations consistent with both global and locally circulating variants underscores the dynamic nature of viral evolution and the necessity for flexible surveillance methods that account for emerging mutations outside traditional variant classifications.

This study has some limitations that should be considered when interpreting the results. Although the large number of masks were analyzed, the sample may not fully reflect the diversity of mask types, usage patterns, and environmental conditions in the general population. Additionally, viral RNA quantification does not equate to viral viability or infectivity, as RNA detection does not confirm the presence of a live virus. Furthermore, the timing and frequency of mask collection varied between individuals, which may have introduced variability in viral load measurements. Sequencing was only successful in a subset of high-load samples, potentially under-representing the full spectrum of circulating variants. Additionally, behavioral variables—such as mask adjustment, speech, or coughing—were not standardized and may have affected the spatial distribution of viral RNA. Moreover, N95 masks were not included in this study, as their use in Brazil during the pandemic was largely limited to higher socioeconomic groups, and were not widely adopted by the public.

Another limitation of this study is the sample size and the recruitment of participants from only a few institutions, which may limit the generalizability of mask-use behaviors and environmental conditions. Storing masks in domestic freezers, even with standardized instructions, may have introduced variability in temperature and humidity that could influence RNA preservation. In addition, restricting sequencing to samples with Ct < 30 likely excluded low-viral-load cases and may have underrepresented circulating variants. Future studies should include larger and more diverse populations, controlled storage conditions, and experimental assessments of RNA stability in different mask materials. These factors may introduce variability in viral detection and should be considered when evaluating the representativeness of our results. Moreover, inter-individual differences in facial anatomy and mask fit can alter airflow trajectories and deposition patterns, contributing to the spatial heterogeneity of viral load observed across mask regions.

Notably, a major strength of this study is the demonstration that masks can serve as a fully non-invasive sampling tool, improving participant acceptability for repeated testing and enabling surveillance at a broader scale in community environments. This approach is particularly advantageous for groups in whom nasopharyngeal swabbing is challenging—such as children, older adults, and individuals with disabilities. Furthermore, mask-based sampling reduces the need for trained personnel and specialized infrastructure, making it a feasible and scalable option for epidemiological monitoring.

## 5. Conclusions

This study provides a comprehensive analysis of SARS-CoV-2 viral load retention in face masks worn by infected individuals, evaluating spatial distribution across nasal, oral, and lateral regions, and comparing structural differences between surgical and fabric masks. Viral load quantification across internal, middle, and outer mask layers, combined with longitudinal data, was used to investigate viral clearance over time. No statistically significant differences in viral load were observed between surgical and fabric masks across any layer or region. Both mask types exhibited similar performance in the internal, middle, and outer layers of the nose and mouth, and in the lateral sections, with comparable viral loads and high inter-individual variability. Longitudinal analysis revealed a consistent and statistically significant reduction in viral load over time, with an estimated daily decrease of 0.216 log_10_ units, equivalent to a 39% decline per day. This trend was confirmed through both an LMM and an exponential decay fit (R^2^ = 0.88), supporting the hypothesis of progressive viral clearance through exhalation. Collectively, these findings highlight that both surgical and fabric masks are similarly effective in retaining viral particles on their surface, and that viral contamination of masks decreases substantially with time, reinforcing current recommendations on mask usage duration and hygiene. Furthermore, this study demonstrated the possibility of amplifying and sequencing SARS-CoV-2 genetic material directly from used face masks. This finding reinforces the potential of masks, a readily available and non-invasive source of viral RNA, as a complementary tool for genomic surveillance and monitoring of SARS-CoV-2 circulation in the community. Such an approach could provide valuable epidemiological insights, especially in scenarios where conventional sampling methods, such as nasopharyngeal swabs, are limited or not feasible. Future studies should evaluate whether this mask-based genomic surveillance approach can be extended to other respiratory viruses with similar transmission dynamics.

## Figures and Tables

**Table 1:** Oligonucleotides (primers) were used for amplification and sequencing reactions of the partial gene of the SARS-CoV-2 spike protein in this study.

Primer ID	Sequence (5′-3′)	Positions *	Strand	Function
RT-PCR-F1	ACCCTGACAAAGTTTTCAGATCCT	21,675 (113)–21,698 (136)	forward	RT PCR
RT-PCR-R1	CCTGATAAAGAACAGCAACCTG	23,402 (1819)–23,381 (1840)	reverse	RT PCR
PCR-F2	TTCAACTCAGGACTTGTTCTTACC	21,709 (147)–21,732 (170)	forward	Nested PCR and sequencing
PCR-R2	GTGGATCACGGACAGCATC	23,300 (1720)–23,282 (1738)	reverse	Nested PCR and sequencing
SEQ-F2	TTGGATGGAAAGTGAGTTCAGA	22,036 (453)–22,015 (474)	forward	sequencing
SEQ-F3	TTGTTTAGGAAGTCTAATCTCAAACC	22,925 (1363)–22,950 (1388)	forward	sequencing
SEQ-R1	GCTGAGAGACATATTCAAAAGTGCA	22,082 (496)–22,058 (250)	reverse	sequencing
SEQ-R2	GTGTGCTACCGGCCTGATAG	22,978 (1416)–22,997 (1435)	reverse	sequencing

* Position of primers in the genome and in the Spike protein gene in parentheses. Reference genome NC45512.2 (Wuhan). All oligonucleotide sequences are written in sense orientation in this table. Adapted from La Rosa and colleagues in 2021 [25].

**Table 2:** Means ± standard deviation of the viral load of each mask region.

Region	Mean ± SD	Layer	Mean ± SD
Nose	2.43 × 10^6^ ± 1.93 × 10^7^	Intern	6.50 × 10^6^ ± 4.84 × 10^7^ *
		Center	2.84 × 10^6^ ± 2.11 × 10^7^
		Extern	4.25 × 10^5^ ± 2.65 × 10^6^
Mouth	1.17 × 10^6^ ± 9.28 × 10^6^	Intern	2.67 × 10^6^ ± 2.61 × 10^7^ **
		Center	9.38 × 10^5^ ± 6.25 × 10^6^
		Extern	3.38 × 10^4^ ± 1.91 × 10^5^
Side	7.07 × 10^4^ ± 7.17 × 10^5^	Right	1.23 × 10^5^ ± 1.43 × 10^6^
		Left	1.86 × 10^4^ ± 6.72 × 10^4^

Mean ± standard deviation of the cut’s masks in the regions of nose, mouth, and sides and also the layers of these same regions, as follows: Inner; Central; External; Right and Left. * *p* < 0.05 in the comparison test internal × central Layer. ** *p* < 0.05 in the comparison test internal × central layer, internal × external layer.

**Table 3:** Means ± standard deviation (SD) of the cuts of the masks in the nose, mouth, and sides from two different mask types: fabric and surgical.

Type	Region		Mean ± SD
Fabric	Nose	Internal	2.47 × 10^5^ ± 1.12 × 10^6^
		Central	1.12 × 10^6^ ± 5.05 × 10^6^
		External	3.46 × 10^4^ ± 1.82 × 10^5^
	Mouth	Internal	7.61 × 10^5^ ± 5.25 × 10^6^
		Central	1.04 × 10^4^ ± 3.77 × 10^4^
		External	5.09 × 10^4^ ± 2.38 × 10^5^
Surgical	Nose	Internal	1.17 × 10^7^ ± 6.53 × 10^7^
		Central	3.50 × 10^6^ ± 2.46 × 10^7^
		External	7.50 × 10^5^ ± 3.56 × 10^6^
	Mouth	Internal	4.26 × 10^6^ ± 3.51 × 10^7^
		Central	1.28 × 10^6^ ± 7.30 × 10^6^
		External	2.77 × 10^4^ ± 1.38 × 10^5^

Mean ± standard deviation of the cut’s masks in the regions of nose and mouth and also the layers of these same regions, as follows: Inner; Central and External from fabric and surgical masks. Although numerical differences were observed between mask types, no statistically significant differences were detected in any region or layer.

## Data Availability

The clinical and demographic data of participants used in this study are confidential and cannot be shared due to ethical restrictions. However, partial SARS-CoV-2 spike gene sequences obtained in this study have been deposited in GenBank under accession numbers PV797855, PV797856, PV797857, PV797858, PV797859, PV797860, PV797861, PV797862, PV797863, PV797864, PV797865, and PV797866.

## Abbreviations

The following abbreviations were used in this manuscript:

SARS-CoV-2	Severe Acute Respiratory Syndrome Coronavirus 2
COVID-19	Coronavirus Disease 2019
RT-qPCR	Reverse transcription quantitative polymerase chain reaction
VOC	Variant of Concern

## Appendix A

**Table A1:** Epidemiological and genetic analysis of individuals with sequenced SARS-CoV-2 samples.

ID	Date	Gene E (sar-Cov-2)		Symptoms	Age	Comorbidities	Sex	Variant	Deletion	Substitutions in RBD			
		**Ct**	**Copy/mL**							**K417N/T**	**L452R**	**E484K/A**	**N501Y**
PV797855	06/22/2022	18	2.80 × 10^9^	Unknown	Unknown	Unknown	Female	Omicron	+	N	R	A	Y
PV797858	05/10/2022	17.14	5.10 × 10^9^	Unknown	Unknown	Unknown	Male	Omicron	-	N	L	A	Y
PV797859	05/10/2022	23.27	7.06 × 10^7^					Omicron	-	N	L	A	Y
PV797856	09/25/2021	14.98	2.30 × 10^10^	cough and runny nose	38	Pregnant women and gestational diabetes	Female	Delta	+	K	R	E	N
PV797857	09/25/2021	20.48	4.95 × 10^8^					Delta	+	K	R	E	N
PV797860	12/04/2020	21.55	2.35 × 10^8^	Unknown	29	Unknown	Male	Clade 19B	-	K	L	E	N
PV797861	12/04/2020	20.11	6.41 × 10^8^	Unknown	41	Unknown	Female	Clade 20B	-	K	L	K	N
PV797862	03/01/2021	15.13	2.07 × 10^10^	Unknown	28	Unknown	Female	Gamma	-	T	L	K	Y
PV797863	03/01/2021	25.61	1.38 × 10^7^					Gamma	-	T	L	K	Y
PV797864	03/30/2021	14.39	3.47 × 10^10^	Sore throat, cough and body ache	33	Unknown	Female	Gamma	-	T	L	K	Y
PV797865	07/08/2021	24.46	3.08 × 10^7^	headache and throat	34	pregnant	Female	Gamma	-	T	L	K	Y
PV797866	07/08/2021	29.35	1.01 × 10^6^	headache, throat and cough	80	Unknown	Male	Gamma	-	T	L	K	Y

The table presents key epidemiological characteristics of individuals whose samples underwent sequencing, it also presents the variants of concern, if the sequence presents any deletions or amino acid substitutions when present (in red). ID: Sample identification; A—Alanine; E—Glutamic Acid; K—Lysine; N—Asparagine; T—Theonine; R—Arginine; L—Leucine and Y—Tyrosine.
